# Using de novo genome assembly and high-throughput sequencing to characterize the MHC region in a non-model bird, the Eurasian coot

**DOI:** 10.1038/s41598-022-11018-w

**Published:** 2022-04-29

**Authors:** Ewa Pikus, Piotr Minias

**Affiliations:** Department of Biodiversity Studies and Bioeducation, Faculty of Biology and Environmental Protection, University of Łódź, Banacha 1/3, 90-237 Łódź, Poland

**Keywords:** Evolution, Genetics, Immunology

## Abstract

Genes of the Major Histocompatibility Complex (MHC) form a key component of vertebrate adaptive immunity, as they code for molecules which bind antigens of intra- and extracellular pathogens (MHC class I and II, respectively) and present them to T cell receptors. In general, MHC genes are hyper-polymorphic and high MHC diversity is often maintained within natural populations (via balancing selection) and within individuals (via gene duplications). Because of its complex architecture with tandems of duplicated genes, characterization of MHC region in non-model vertebrate species still poses a major challenge. Here, we combined de novo genome assembly and high-throughput sequencing to characterize MHC polymorphism in a rallid bird species, the Eurasian coot *Fulica atra*. An analysis of genome assembly indicated high duplication rate at MHC-I, which was also supported by targeted sequencing of peptide-binding exons (at least five MHC-I loci genotyped). We found high allelic richness at both MHC-I and MHC-II, although signature of diversifying selection and recombination (gene conversion) was much stronger at MHC-II. Our results indicate that Eurasian coot retains extraordinary polymorphism at both MHC classes (when compared to other non-passerine bird species), although they may be subject to different evolutionary mechanism.

## Introduction

The Major Histocompatibility Complex (MHC) is a complex multi-gene family playing an essential role in vertebrate adaptive immune response. The MHC genes code for key transmembrane molecules recognizing and binding foreign peptides in order to present them to immune cells, which initiates an immune response^1,2^. Two major classes of MHC genes can be distinguished, including class I genes (MHC-I) which are expressed on the surface of almost all nucleated somatic cells, and class II genes (MHC-II) which are expressed on the specialized cells of the immune system, such as B lymphocytes and macrophages^3^. Antigens recognized by MHC-I molecules originate from intracellular pathogen proteins processed via proteasomal proteolysis. They are presented to T CD8 + lymphocytes, resulting in a direct destruction or the initiation of apoptotic processes within infected cells. In turn, exogenous antigens derived from extracellular pathogens processed by endocytosis or autophagy are presented by MHC-II molecules to T CD4 + lymphocytes. This allows for the trigger of a proper immune response pathway^4,5^. Antigens recognized by MHC-I and MHC-II comprise small peptides composed of 8–10 and 15–20 amino acid residues, respectively, and they are bound by the most polymorphic region of MHC molecules, the peptide-binding groove^5^. The peptide-binding region (PBR) of any particular MHC allelic variant can bind a limited number of foreign peptides and, hence, the number of different MHC molecules expressed in the body determines an array of pathogens recognizable by the mechanisms of an adaptive immune defence^6^.

An extreme polymorphism within the area of the MHC peptide-binding groove may be considered one of key evolutionary features of these genes. Due to host–pathogen arms race, MHC-I and MHC-II genes demonstrate the highest level of functional polymorphism known in vertebrates^3,7,8^. For example, thousands of different alleles were identified in the global human population at some MHC loci^9^. As for birds, the sedge warbler *Acrocephalus schoenobaenus* with over 3500 MHC-I alleles^10^ and common yellowthroat *Geothlypis trichas* with almost 1000 MHC-II alleles recorded within a single population^11^ may provide examples of species with exceptionally high MHC polymorphism. It is possible to distinguish three basic mechanisms of natural (balancing) selection responsible for the maintenance of MHC polymorphism in vertebrate populations. First, the mechanism of heterozygote advantage assumes that heterozygous individuals are able to recognize a broader scope of antigens, which should provide them with fitness benefits over homozygotes, especially when exposed to diverse pathogen faunas^12,13^. Second, parasites or pathogens may evolve quickly to avoid host immune defences. In this scenario, negative frequency‐dependent selection can maintain MHC polymorphism via favouring rare alleles (rare-allele advantage hypothesis). Alleles that increase in frequency gradually lose their selective advantage, as pathogens tend to evolve towards the avoidance of the most common immune barriers of their hosts (including the most frequent MHC alleles)^13,14^. Third, high polymorphism of MHC genes in populations may be generated and maintained in response to pathogen-driven selection that varies in space and time (fluctuating selection)^15^. All these mechanisms are expected to produce an apparent excess of non-synonymous (amino acid altering) mutations (dN) over synonymous (silent) mutations (dS) within the region of the peptide-binding groove. Thus, high values of dN/dS ratio (>1) provide a molecular signature of balancing selection acting on the MHC^16,17^.

Extensive gene duplication constitutes the next characteristic feature of MHC genes in birds^18^. For instance, the number of gene copies has been reported to vary between 1 and 33 for MHC-I and 1–23 for MHC-II^19^. However, the latest genomic studies indicate that duplication rate in some avian evolutionary lineages may be even greater^20^. Duplicated genes may retain similar molecular features or even share identical alleles, which is mainly due to the processes of concerted evolution, where different loci evolve non-independently, leading to their homogenization within species^21–23^. This triggers methodological difficulties in the MHC genotyping, pertaining particularly to difficulties in designing locus-specific primers in non-model taxa. A common usage of conserved (multi-locus) primers preclude application of traditional Sanger sequencing methods for MHC genotyping and only after the development of new generation sequencing (NGS) methods a decisive breakthrough in the MHC studies took place^24^. Another milestone in the MHC research was reached via development of techniques used for the sequencing and assembling high-quality genomes. Genomic approaches are not restricted to sequencing single exons (as it frequently is with high-throughput MHC genotyping at the population scale), but provide resolution of complete genes, or may even provide information on the architecture of the entire MHC region^20^.

The aim of our paper was to characterize the MHC region in a non-model species of a wild bird, the Eurasian coot *Fulica atra*. This is a medium-size non-passerine waterbird from the rail family (Rallidae), which has a broad geographical range (spanning from Europe through Asia to Australia) and a large global population of ca. 5.3–6.5 million individuals^25^. We used both standard high-throughput sequencing (NGS) to characterize polymorphism and selection at the key MHC exons coding for PBR (within Central European population), as well as genomic techniques to characterize the architecture of the MHC region. To date, polymorphism of MHC-II has already been examined in the Iberian population of our study species using targeted sequencing of a single PBR exon (no genomic resources available for this population)^26^. Therefore our primary aim was to comprehensively characterize the MHC-I genes and to compare the levels of polymorphism and signature of selection between both MHC classes, as they may show distinct evolutionary trajectories in birds^27^.

## Material and methods

### Sample collection

Fieldwork took place in central Poland, mostly in the city of Łódź (51° 45′ N, 19° 28′ E) and non-urban areas located nearby. Blood samples were collected from adult birds (*n* = 283) captured mostly during the reproductive season (March-July) between 2012 and 2019. We caught birds at nests or while feeding on the shore using noose traps made from monofilament nylon. All birds were ringed with metal rings (tarsus) and plastic collars (neck) to enhance identification of individuals in the field and avoid recaptures. From each captured bird we took 50 μl of blood from a tarsal vein into 96% ethanol and stored the samples in 5 °C until DNA isolation. We extracted genomic DNA using GeneJET Genomic DNA Purification Kit (Fermentas, Thermo Fisher Scientific, Waltham, MA, USA) according to the manufacturer’s protocol. Bird capturing and blood sampling was performed by the permissions of the Local Bioethical Commission for Experiments on Animals in Łodź (nos 40/ŁB 620/2012 and 15/ŁB/2016) and complied with current laws of Poland Act on Nature Conservation from 16 April 2004 (Journal of Laws from 2004, No. 92, item 880). All reporting follows the recommendations in the ARRIVE guidelines.

### Genome sequencing and assembly

To get an insight into the architecture of the MHC region (gene copy numbers) and polymorphism of non-PBR exons in the Eurasian coot we generated de novo genome assembly for a single individual from our study population. For this purpose, one Chromium linked-read library was constructed with the Chromium Genome Library Kit & Gel Bead Kit v2 (10 × Genomics, Pleasanton, CA, USA) and sequenced on an SP lane on an Illumina NovaSeq 6000 instrument (Illumina Inc., San Diego, CA, USA) at the Carver Biotechnology Center at the University of Illinois at Urbana-Champaign. A total of 889,998,744 paired-end reads (2 × 150nt) were generated, demultiplexed, and assembled with Supernova v2.1.1 (10 × Genomics), setting maximum reads used to *n* = 600,000,000 and all other parameters as default. Supernova output was converted to *pseudohap* FASTA format for downstream processing. The genome assembly was filtered for duplicate contigs and scaffolds using the *dedupe* script from BBMap v38.36^28^. A custom vector-screening script was employed to remove residual sequencing adapters and vector sequence. No contaminant non-bird sequences were detected in the genome assembly as assessed by BlobTools v0.9.19.6^29^. Contigs and scaffolds less than 1 kb were filtered from the assembly prior to acceptance at NCBI under BioProject PRJNA633903 and GenBank accession GCA_013372525.1. Genome completeness was estimated at 93.3% (1.3% duplicated), as assessed by BUSCO v3.0.1^30^ using the Aves odb9 lineage of 4,915 orthologs. Total sequence length of the genome assembly was 1168 Mb, scaffold N50 was 6.4 Mb, while contig N50 was 0.25 Mb.

The genome assembly was annotated by employing three rounds of MAKER v3.01.1^31^. In the first round of MAKER annotation, gene models were predicted using homology searches from the following lines of evidence: transcriptome assembly sequences from NCBI for Okinawa rail *Gallirallus okinawae* (ICPP01000000) and laughing gull *Leucophaeus atricilla* (GFNV00000000), as well as proteins from available NCBI RefSeq sequences (Gruiformes) and the SwissProt database. Two MAKER ab initio gene predictors, SNAP and Augustus, were trained using gene models predicted from comparative evidence output from the first round. A second round of MAKER utilized the trained models for Augustus and SNAP ab initio gene prediction. A final third round of gene prediction used re-trained models for SNAP and Augustus from the second round of output.

### Retrieving MHC sequences from genomic data

To retrieve MHC class I and class II from genome assembly we performed BLAST searches using available non-passerine MHC sequences. First, to retrieve contigs with MHC we BLASTed concatenated exonic sequences containing exons 1–5 for MHC-I and exons 1–4 for MHC-II. The remaining exons were not included because of short length (< 35 bp). For both MHC classes we used available GenBank sequences from the Chinese egret *Egretta eulophotes* (KY511591 and KC282841). We also checked blasting results using sequences from other non-passerine species (KC282841 from red knot *Calidris canutus* and KC205115 from golden pheasant *Chrysolophus pictus* for MHC-I; HM070250 from mallard *Anas platyrhynchos* and AB872444 from crested ibis *Nipponia nippon* for MHC-II), but they produced very consistent results. Second, we aimed to retrieve all available PBR sequences, which are coded by exons 2 and 3 of the same gene at MHC-I and by exons 2α and 2β coded by separate genes at MHC-II^32,33^. For this purpose, we first retrieved PBR exon sequences from longer MHC contigs and used them to repeat blastn searches (a single exon per search) within the genome assembly.

### MHC genotyping (high-throughput sequencing)

To get a better resolution of MHC polymorphism and the mechanisms that may contribute to its maintenance (recombination and selection) in the Eurasian coot, we genotyped key MHC regions (selected PBR-coding exons) in all captured individuals (283 individuals genotyped at MHC-I, 230 genotyped at MHC-II). Population genotyping focused on a single exon per MHC class (exon 3 at MHC-I and exon 2β at MHC-II), as these exons are traditionally targeted in avian MHC research (allowing direct comparisons across species) and their polymorphism is expected to be well representative for the entire PBR region^34^. To genotype MHC-I exon 3, we used primers MHCI-int2F (5’-CATTTCCCTYGTGTTTCAGG-3’) and MHCI-ex4R (3’-GGGTAGAAGCCGTGAGCRC-5’), which were originally designed for accipitrid birds^35^. Primer MHCI-int2F binds to the conserved flanking region of intron 2 and primer MHCI-ex4R binds to the conserved region of exon 4. *S*pecificity of these primers towards coot MHC-I genes was verified using our genome assembly, showing no mismatches within the 3-terminus region, which is crucial for effective PCR amplifications^36^. Consequently, non-specific MHC-I amplifications (allele drop out) were unlikely. The length of the entire amplicon was 411 bp, including almost entire exon 3 (273 bp out of 276 bp). Species-specific primers Fuat-Ex2Fw (5′-CTGACCRGCCTCCCTGCA-3′) and Fuat-Ex2Rv (5′-TTGTGCCAYACACCCACC-3′) were used to amplify MHC-II. These two primers were originally designed for the Eurasian coot^26^ and they successfully amplify the entire MHC-II exon 2 (270 bp), binding to the flanking regions of intron 1 and 2. In each PCR reaction we used fusion primers with Illumina Nextera Transposase adapter sequences (Illumina Corp., San Diego, CA, USA) and 7-bp barcodes to identify the samples. PCR amplifications were carried out in a final volume of 20 μl containing 20–80 ng genomic DNA (1 µl of DNA isolate), 10 µl of 2X HotStarTaq Plus MasterMix Kit (Qiagen, Venlo, The Netherlands), 8 µl of deionized water and 0.5 µl of each primer. PCR protocols followed Alcaide et al.^35^ for MHC-I and Alcaide et al.^26^ for MHC-II, although in both cases the number of PCR cycles was reduced to 25 to suppress the formation of artificial chimeras, which could confound the correct interpretation of Illumina sequencing results. The effects of PCR reactions were confirmed for each sample by visual examination of band intensities on 2% agarose gel electrophoresis. To purify PCR products we used AMPure XP magnetic beads (Beckman Coulter, Brea, CA, USA) and concentration estimates were quantified using Quant-iT PicoGreen dsDNA marking kit (Thermo FisherScientific, Waltham, MA, USA). Separate libraries for MHC class I and II were prepared using equimolar concentrations of purified PCR products and NEB-Next DNA Library Prep Master Mix Set for Illumina (New England Biolabs, Ipswich, MA, USA). Both libraries were sequenced on the 2 × 250 bp Illumina MiSeq platform.

In the processing of raw Illumina data we used an online webserver, the Amplicon Sequencing Analysis Tools (AmlpiSAT)^37^, and followed recommendations by Biedrzycka et al.^38^. In the first step we used the Amplicon Sequencing MERGing (AmpliMERGE) tool, which merges paired-end reads, optimizing their overlapping lengths according to amplicon data^39^. Next, we used the Amplicon Sequencing Assignment (AmpliSAS) tool, which performs read demultplexing, variant clustering and putative allele filtering based on user-specified criteria. For the clustering step (identification of reads resulting from genotyping errors and clustering them with reads identified as true alleles) we used default AmpliSAS settings for Illumina data, including a substitution error rate of 1%, an indel error rate of 0.001% and the minimum dominant frequency of 25%. Finally, we used AmpliSAS to filter for clusters that are likely to be artefacts, including chimeras and other low-frequency artefacts (>3%) that were retained through the clustering step. Samples with amplicon depth of less than 300 reads were excluded from the analyses and the maximum amplicon depth was, by default, set to 5000 reads because of AmpliSAS performance reasons. The average amplicon depth prior to the processing was 4453 ± 66 [SE] reads for MHC-I and 2616 ± 97 [SE] reads for MHC-II. We obtained validated MHC-I and MHC-II genotypes for 270 and 220 individuals, respectively. Technical reproducibility of validated sequences was 93.7%, as estimated using 36 technical replicates (i.e. samples for which two amplicons were obtained in independent PCR reactions and sequenced). To align all unique MHC class I and II sequences we used Geneious v10.0.5 (Biomatters Ltd., Auckland, New Zealand). We removed intron regions from the alignments and we inferred alleles based on the exon fragments only.

### Recombination

Recombination signal at the MHC-I exon 3 and MHC-II exon 2 (NGS data) was searched for using RDP v.4.97 software, which implements different algorithms developed specifically to detect recombinant sequences^40^. We used seven basic algorithms (Maxchi, BootScan, Genconv, SiScan, RDP, Chimaera, and 3Seq) and ran all the analyses using default settings with statistical significance threshold of *P* = 0.05 and Bonferroni correction for multiple comparisons. To quantify recombination signal we calculated the number of different recombination events, number of recombinant sequences, and number of breakpoints within 100 nucleotide window. A recombination event was recognized when supported by two or more algorithms, while events recognized by a single algorithm were discarded. Presence of recombination hot and cold spots was tested with the local hot/cold-spot test (1000 permutations), as implemented in RDP software.

### Sequence polymorphism and selection

We used DnaSP v.6.10.3 software^41^ to characterize MHC class I and II polymorphism (NGS and genome assembly data). We assessed sequence polymorphism as the number of polymorphic sites, total number of mutations, average nucleotide diversity, and average number of nucleotide differences. To quantify the signature of selection at the MHC-I exon 3 and MHC-II exon 2 (NGS data) we calculated the dN/dS ratios, which reflect the relative rate of nonsynonymous (amino acid altering) to synonymous (silent) nucleotide substitutions (per non-synonymous and synonymous site, respectively). Positive (diversifying) selection is detected when new allelic variants are promoted, which means that nonsynonymous substitutions accumulate faster than synonymous substitutions (dN/dS > 1) and similar pattern is expected under pathogen-driven balancing selection, when multiple alleles are maintained within populations. In contrast, negative (purifying) selection removes most nonsynonymous substitutions, which thus accumulate more slowly than synonymous ones (dN/dS < 1). Finally, similar rates of nonsynonymous and synonymous substitutions (dN/dS ≈ 1) indicate neutral evolution and no detectable signature of selection. We measured codon-specific signature of positive (diversifying) and negative (purifying) selection using two approaches, Bayesian inference (Fast Unconstrained Bayesian AppRoximation, FUBAR) and maximum likelihood (Fixed Effects Likelihood, FEL), implemented in HyPhy software available at the Datamonkey webserver^42^. We used 0.95 posterior probability (FUBAR) and P < 0.05 (FEL) thresholds to identify sites that may have experienced pervasive (apparent across all alleles) diversifying or purifying selection. We also used Mixed Effect Model of Evolution (MEME) to identify sites subject to episodic (apparent across a subset of alleles) diversifying selection (P < 0.05). We used default settings and input trees inferred from alignments in all the analyses. Selection analyses were performed on alignments lacking recombinant sequences, because recombination can mask true phylogenetic relationships between allelic variants (allele tree topology) and, thus, lead to erroneous estimates of the nucleotide substitution rates^43^. Positions of positively selected sites were compared with putative PBR sites in non-passerine birds (as identified based on the global analysis of selection at the avian MHC^27^) and humans (based on the crystallographic structure of MHC molecules^44,45^). Positions of positively selected sites at MHC-II exon 2 were also compared with previous data from Iberian coot population^26^. To quantitatively assess an agreement between these positions we calculated intra-class correlation (ICC) coefficients in the *irr* R package^46^.

## Results

### Genome assembly

Our BLAST searches of MHC-I exons 1–5 retrieved 32 contigs containing all five exons and another 12 contigs containing four exons (either 1–4 or 2–5). Visual inspection of these sequences retained 27 functional haplotypes, out of which 21 contained exons 1–5 (Fig. S1 in the Electronic Supplementary Material). All five exons showed similar level of polymorphism, although exon 4 had noticeably lower nucleotide diversity from the remaining exons (Table 1). Also, the number of retrieved haplotypes (*n* = 27) suggested the presence of over ten duplicated MHC-I loci in the Eurasian coot. However, when we BLASTed only exon 2 or exon 3 against genome assembly, we retrieved 76 and 82 unique functional alleles, respectively. Although it is likely that many of these sequences may represent genotyping artefacts, overall they seem to provide support for high duplication rate at MHC-I in our study species. BLAST searches for MHC-II exons 1–4 retrieved only one contig containing a single sequence of α and β chain (Fig. S1). Using PBR exons for blast searches yielded similar results (one sequence of each exon retrieved).

**Table 1:** Polymorphism of MHC class I and MHC class II in the Eurasian coot, as inferred from genome assembly and high-throughput (Illumina sequencing).

MHC	Method	Exon	Length (bp)	No. sequences	No. segregating sites	Total no. mutations	Nucleotide diversity
Class I	Genome assembly	1	64	25	38	56	0.158
		2	264	27	130	160	0.107
		3	276	27	131	166	0.122
		4	273	27	90	103	0.051
		5	105	23	43	54	0.116
	Illumina sequencing	3	273	165	181	251	0.103
Class II	Illumina sequencing	2	270	147	103	143	0.118

### Polymorphism of PBR exons

**Figure 1:**
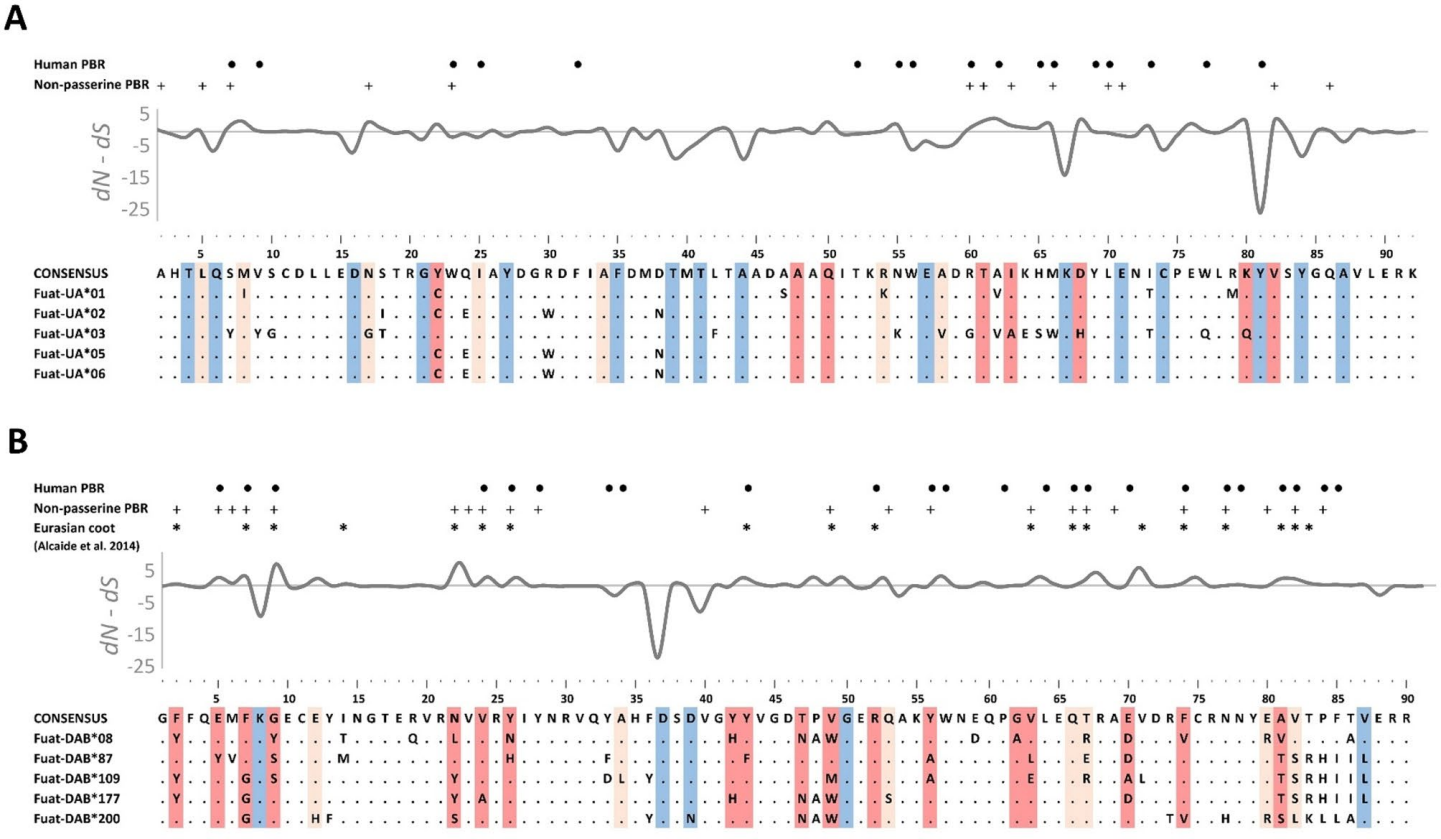
Alignments of amino acid sequences of MHC class I exon 3 (**A**) and MHC class II exon 2 (**B**) in the Eurasian coot. Five most frequent variants of each exon are shown. Dots indicate amino acids identical with the reference consensus sequence. Sites under pervasive and episodic positive selection are marked with dark and light red, sites under pervasive negative selection are marked with blue. Pervasive and episodic selection was assessed with FEL/FUBAR and MEME, respectively, using non-recombinant sequences only. Variation in selection parameter (*dN* – *dS*) is shown above the alignments. Residues of the putative peptide-binding region (PBR) in non-passerine birds (based on the global analysis of selection at the avian MHC by Minias et al.^27^) and humans (based on the crystallographic structure of MHC molecules by Saper et al.^44^ and Brown et al.^33^) are indicated with crosses ( +) and large dots (·) at the top of each panel. Positively selected sites previously recognized at MHC class II exon 2 by Alcaide et al.^26^ are indicated with asterisks (*).

**Figure 2:**
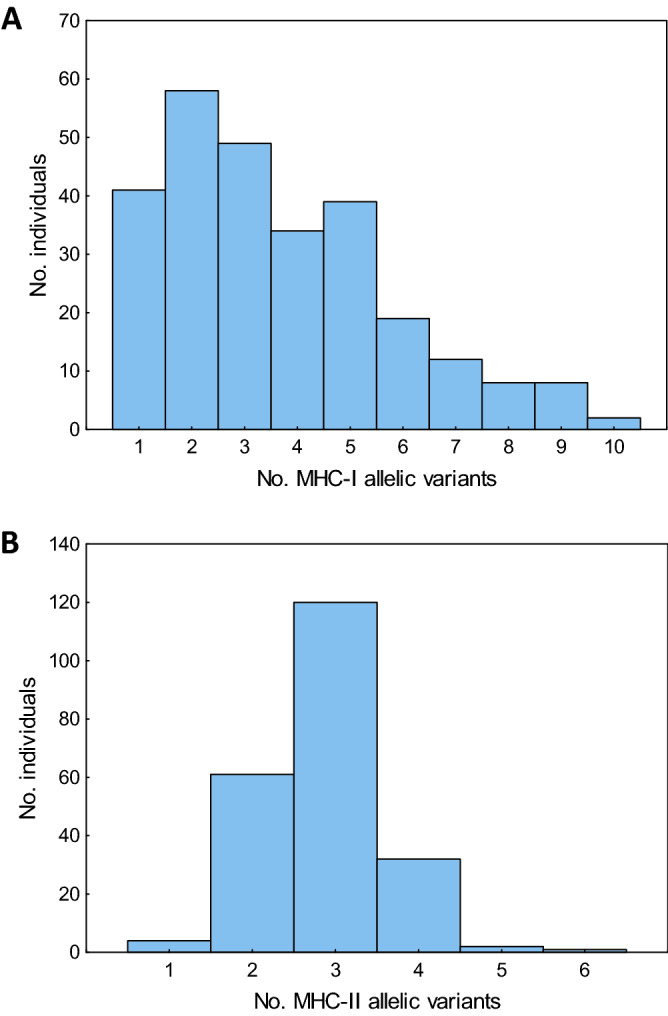
The number of MHC class I (**A**) and MHC class II (**B**) allelic variants recorded per individual in the Eurasian coot.

High-throughput sequencing of PBR exons revealed high level of allelic richness in our study coot population, as in total we detected 165 allelic variants of MHC-I exon 3 (*n* = 270 individuals genotyped) and 147 allelic variants of MHC-II exon 2 (*n* = 220 individuals) (Table 1). All allelic variants were functional (no stop codons or frameshift mutations), providing no evidence of pseudogenization. We recorded a relatively minor frequency (14.3%) of variants with an indel mutation at MHC-II exon 2 (one-codon deletion at position 84, as marked at Fig. 1), but they all retained functionality. The maximum number of allelic variants recorded per individual was ten at MHC-I and six at MHC-II, indicating that we genotyped at least five MHC-I and three MHC-II loci. Most frequently we recorded two MHC-I and three MHC-II variants per individual (21.5% and 44.4%, respectively) (Fig. 2). The total number of segregating sites and total number of mutations were higher at MHC-I exon 3, but MHC-II exon 2 had slightly higher nucleotide diversity, suggesting similar levels of polymorphism at MHC class I and II.

### Recombination

**Figure 3:**
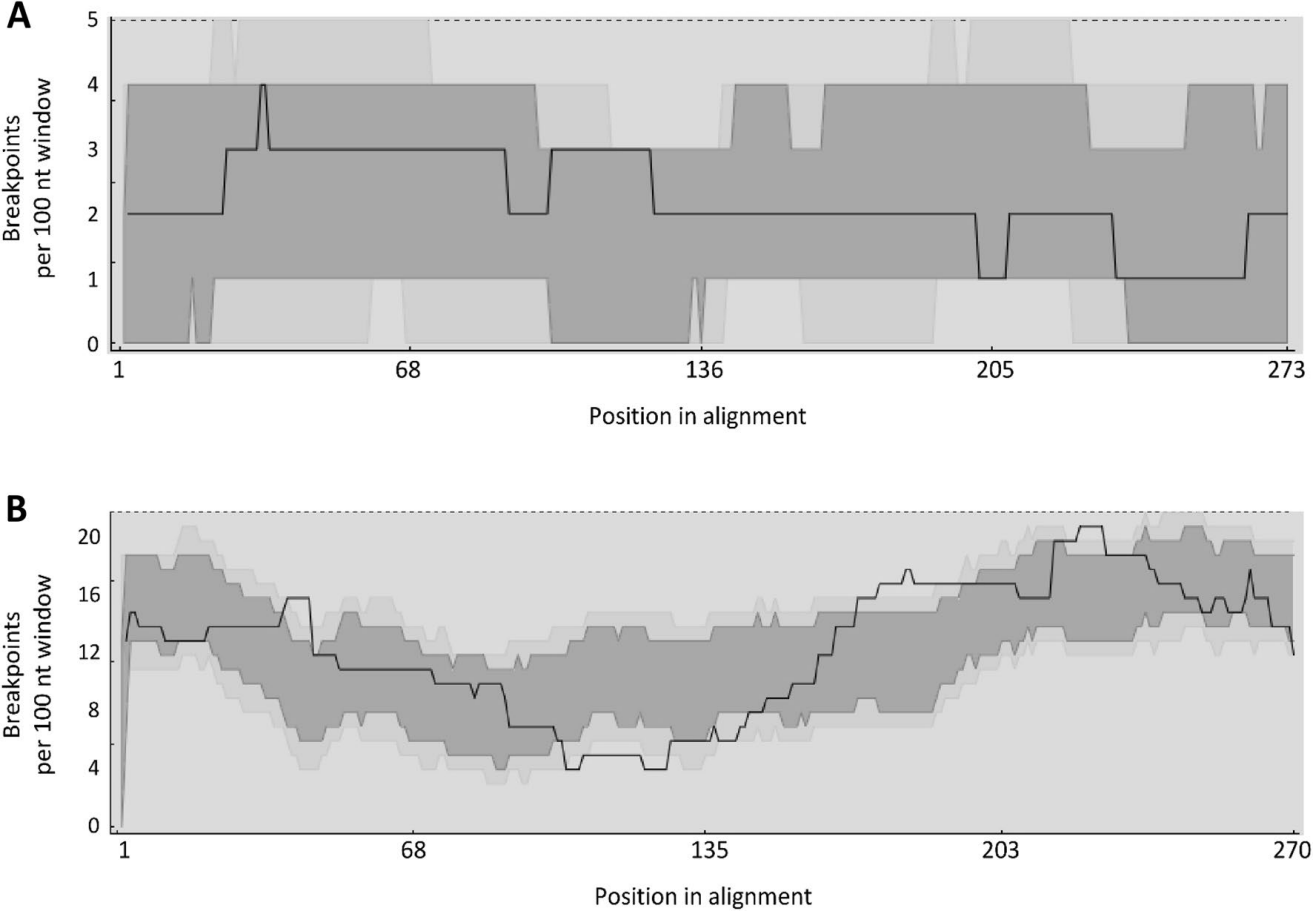
Recombination signal at the MHC class I exon 3 (**A**) and MHC class II exon 2 (**B**). Black line indicates the number of breakpoints detectable per 100 nucleotide (nt) window. Dark and light grey areas indicate the 95% and 99% confidence intervals for the expected degrees of breakpoint clustering in the absence of recombination hot- and cold-spots (as assessed with the local hot/cold spot test). Positions in alignment where black line emerges above the grey areas indicate a recombination hot-spot, while positions where it drops below the grey areas indicate a recombination cold-spot.

We found evidence for much stronger recombination signal at the MHC class II than class I. At MHC-I we identified three recombination events and the total number of recombinant sequences was 31 (18.8% of all sequences). In contrast, there were 17 recombination events recognized at MHC-II and the number of recombinant sequences was over twice higher (*n* = 68, 45.9% of all sequences). The mean number of breakpoints per 100 nucleotide window ranged from 1 to 4 at the MHC-I, and from 4 to 21 at the MHC-II (Fig. 3). Consistently, three recombination hotspots were detected at MHC-II, while none was detected at MHC-I (Fig. 3).

### Selection

Our analyses provided evidence for much stronger positive (diversifying) selection at MHC-II than MHC-I in the Eurasian coot. Bayesian methods (FUBAR) identified 15 sites under pervasive diversifying selection within MHC-II exon 2, whereas only 7 sites were recognized as under pervasive diversifying selection within MHC-I exon 3 (Table 2, Fig. 1). Maximum-likelihood approach (FEL) provided similar results (17 and 8 sites pervasive diversifying selection at MHC class II and class I, respectively). The number of sites under episodic diversifying selection (MEME) was also higher at MHC-II (*n* = 22) than MHC-I (*n* = 13) (Table 2, Fig. 1). At the same time, the number of sites under negative (purifying) selection was much lower at MHC-II when compared with MHC-I (5 vs. 16 sites, Table 2). Stronger diversifying selection at MHC-II than MHC-I was also inferred from the analysis of nucleotide substitution rates (dN/dS), as measured across putative PBR sites recognized in non-passerine birds (2.83 vs. 1.73) or humans (3.37 vs. 1.64) (Table 2). Positions of positively selected sites at MHC-II showed a moderately high agreement with positions of putative PBR sites in non-passerines (ICC = 0.55) and humans (ICC = 0.47), and with positions of sites previously identified as under positive selection in the Iberian coot population (ICC = 0.59) (all *P* < 0.001). Agreement rate at MHC-I was much lower (ICC = 0.28, *P* = 0.003 for putative non-passerine PBR) or non-significant (*P* = 0.90 for human PBR).

**Table 2:** Selection signature at the MHC class I exon 3 and MHC class II exon 2 in the Eurasian coot. PBR indicates putative peptide-binding region.

MHC	Exon	No. of sites			Nucleotide substitution rates (*dN*/*dS*)		
		Pervasive negative selection (FUBAR/FEL)	Pervasive positive selection (FUBAR/FEL)	Episodic positive selection (MEME)	All sites	Non-passerines PBR	Human PBR
Class I	3	13/16	7/8	13	1.702	1.73	1.64
Class II	2	4/5	15/17	22	0.932	2.83	3.37

## Discussion

In order to characterize the MHC in a non-model bird species, the Eurasian coot, we have combined two molecular approaches, i.e. genome assembly and high-throughput sequencing at the population level. The analysis of genome sequences revealed the presence of an unexpectedly large number of MHC-I loci, as contrasted to other non-passerine bird species. This finding confirmed previous genomic analyses of MHC architecture in birds, indicating that the genome assembly approach may produce a better resolution of the MHC region compared to the population-wide genotyping of single exons^20^. On the other hand, our screening of MHC polymorphism at the population level provided support for contrasting evolutionary trajectories at both MHC classes in the Eurasian coot. Although we found a similar allelic and nucleotide diversity at MHC-I and MHC II genes, the mechanisms responsible for maintenance of this variation clearly differed, as MHC-II showed stronger signal of positive (diversifying) selection and recombination (gene conversion).

In general, MHC studies in birds demonstrated that most species of non-passerines seem to have a smaller number of both MHC-I and MHC-II loci compared to passerines, in which the rate of MHC gene duplication is considerably greater^19^. Our study indicates that coot genome may contain more MHC-I loci (ca. 10 loci suggested by the analysis genome assembly, at least 5 loci indicated by targeted exon sequencing) than genomes of most non-passerine bird species studied thus far. For example, birds from the Galliformes order (landfowl), such as the domestic chicken *Gallus gallus*, common quail *Coturnix coturnix*, wild turkey *Meleagris gallopavo* and golden pheasant, have long been recognized to have a compact MHC containing between one and three MHC-I genes^47–49^. In fact, the domestic chicken is known to have the most compact MHC region ever reported (the minimal essential MHC), containing only one dominantly-expressed locus at each class^50,51^. A recent broad-scale comparative analysis confirmed that the vast majority (90%) of non-passerine species have only three or fewer MHC loci of a given class^19^. Higher numbers of MHC loci were recorded in non-passerines only exceptionally, e.g. eight gene copies of MHC-I or MHC-II were found in blue petrel *Halobaena caerulea*^52^ and blakiston’s fish owl *Ketupa blakistoni*^53^, while the maximum level of MHC expansion among non-passerines was recorded in tufted duck *Aythya fuligula* and carmine bee-eater *Merops nubicus* (up to 11–12 functional MHC-I loci)^20^. Contrasted with general patterns of MHC architecture in non-passerine birds, the number of MHC-I loci (> 5) in the Eurasian coot seems to be exceptional and further research is needed to examine whether this is a conserved feature of the MHC across rail family.

In contrast to non-passerine birds, passerines often demonstrate considerably larger numbers of MHC genes, with the average numbers of 7.5 MHC-I and 5 MHC-II loci per species^19^. To date, population-wide genotyping of PBR exons in birds has revealed the presence of up to 33 MHC-I loci in the sedge warbler^10^ and 22 MHC-II loci in the common yellowthroat^54^. However, this approach is expected to underestimate the total number of MHC loci, as it assumes heterozygosity at each locus, and reliable information on MHC copy number variation has been obtained for only a handful of model species with relatively simple architecture of the MHC region^48,55,56^. Taking all this into account, we explicitly acknowledge that our estimates of MHC-I gene copy number in the Eurasian coot may not be accurate. In general, duplicated MHC loci in birds are highly homogenized by inter-locus gene conversion (consistently with concerted evolution), which masks gene orthology^23^. As a result of this homogenization, alleles can be shared between loci^57^ and the true number of paralogs cannot be effectively quantified based on indirect methods (such as allele count within individuals, as used in this study)^19^. This pattern much differs from what we observe in mammals, where divergent evolution often maintains MHC loci independently of each other after duplication event^23^, allowing more reliable assignment of alleles into loci and estimation of gene copy numbers. Furthermore, short-read genome assemblies (as used in this study) cannot provide a good resolution of regions with high duplication rates and are prone to assembly errors^20^. Thus, while our study provide convincing evidence for a high number of MHC-I genes in the Eurasian coot (at least five loci using the most conservative approach), we would need more advanced molecular approaches to get a more accurate estimate of MHC gene copy number in this species. Long-read third-generation sequencing (TGS) techniques are promising with this respect, as they allow much more reliable reconstruction of complex MHC regions, even in passerines. For example, recent analyses of TGS-based genomes provided support for rapid MHC expansion in manakins (Pipridae) with up to 180 MHC-II loci recorded in the golden-collared manakin *Manacus vitellinus*^20^.

A relatively large number of MHC loci in the Eurasian coot may suggest that the species has been exposed to greater pathogen-driven selection over its evolutionary history, when compared to other non-passerines. However, the processes of adaptive MHC gene duplications in response to pathogen and parasite pressure may have a complex nature. On one hand, a comparative study conducted across 54 divergent avian species showed that blood parasite diversity negatively covaried with the number of MHC-I loci, suggesting their effective eradication from hosts with broader spectrum of MHC allelic variants^58^. On the other hand, a positive correlation between the MHC-II gene copy number and helminth richness has been reported in non-passerines, which may reflect an evolutionary (historical) pressure of parasitic faunas on the MHC expansion^59^. It is possible that an apparent discrepancy between these studies is due to different evolutionary trajectories of MHC-I and MHC-II or due to distinct evolutionary processes shaping MHC architecture in major avian lineages (the first study was based mainly on passerines^58^). Irrespective of these differences, pathogens and parasites seem to constitute a leading force that govern the evolution of MHC architecture and duplication processes within this region. At the same time, there are scarcely any studies testing for evolutionary associations between pathogen diversity and MHC copy numbers in vertebrate taxa other than birds. Also, most of this research focused on associations of pathogens or parasites with MHC polymorphism and allelic diversity, rather than with gene copy numbers. For instance, high parasite diversity was associated with greater diversity of MHC-II alleles in some mammalian clades, including rodents, bats and ungulates^60,61^. Despite empirical evidence supporting associations of duplication processes at the MHC with pathogen-driven selection, we acknowledge that pathogen pressure per se seems insufficient to fully explain a huge MHC gene copy number variation, which is observed among divergent vertebrate lineages. Simulation studies indicate that the evolution of MHC numbers may also be driven by the inherent costs of expressing multiple allelic variants^62^, such as the risk of autoimmune diseases or the depletion of T cell receptor (TCR) reservoir^63,64^.

Despite extensive duplications at the MHC-I in the Eurasian coot, our analyses failed to find any convincing evidence for pseudogenization processes. All MHC-I and MHC-II PBR exon sequences retrieved in our study were functional and we found no allelic variants with stop codons or frameshift mutations. Although some haplotypes retrieved from our genome assembly showed the signs of non-functionality, they could be most likely attributed to errors in genotyping or assembly procedures. In accordance with our findings, the previous study on the MHC-II in another (Iberian) population of the Eurasian did not reveal the presence of pseudogenes^26^. In general, the “birth and death” evolution model assumes that over evolutionary times some copies of MHC genes should preserve their primary functions, some others my get duplicated and gain novel functions, while some others turn into pseudogenes via non-functional mutations (e.g. indels)^65,66^. Here, we found a relatively minor frequency of variants with a one-codon deletion at MHC-II exon 2, but they all retained functionality. In general, passerines have much more complex MHC architecture, with many extremely polymorphic and duplicated genes, but also with long introns and pseudogenes. In the great reed warbler *Acrocephalus arundinaceus* 25% MHC-I allelic variants presumably originated from pseudogenes, as they contained a 5-bp deletion in exon 3, which leads to the shift of the reading frame^67^. Similarly, 20% MHC-II sequences were identified as pseudogenic in the red-winged blackbird *Agelaius phoenicus*^68^, while in the house finch *Carpodacus mexicanus* frameshift mutations were recorded in both MHC-II exon 2 and 3^69^. In non-passerines, occurrence of pseudogenes have rarely been reported and non-functional allelic variants, if present, usually showed minor frequencies^70,71^.

Our study showed that MHC expansion in the Eurasian coot was accompanied with high levels of allelic diversity and, in total, we retrieved 165 MHC-I and 147 MHC-II alleles within the Central European population. Even a greater level of the MHC-II allelic polymorphism was previously revealed in the Iberian population of this species (265 alleles), yet these analyses were based on the sample several times greater than ours (902 vs. 283 individuals)^26^. Our findings in combination with previous research^26^ indicate that European coots show the highest degree of MHC-I and MHC-II allelic polymorphism ever reported in non-passerines. Within non-Passeriformes, a relatively high level of MHC polymorphism (though markedly lower than in coots) was found primarily at class II genes. For example, 109 MHC-II alleles were detected in the Mediterranean population of the great flamingo *Phoenicopterus roseus*^72^, and 103 allelic variants were described in a population of lesser kestrels *Falco naumanni*^73^. Yet, most of non-passerine species demonstrate a considerably smaller allelic polymorphism at the MHC (particularly MHC-I) genes. For instance, the MHC-I allelic richness was estimated at 47 alleles in black-tailed godwit *Limosa limosa*^74^, 38 alleles in red-billed gull *Chroicocephalus novaehollandiae scopulinus*^71^ and 36 alleles in red knot^75^. Compared to the Eurasian coot, higher levels of MHC polymorphism have been demonstrated only in passerine birds, reaching hundreds or thousands of alleles in some populations^10,11,76^. A comparison of MHC allelic richness between different coot populations showed that most MHC-II allelic variants (86%) found in our (Central-European) population were previously described in the Iberian population^26^, despite the fact that both populations are spatially separated (the vast majority of our birds do not reach the Iberian Peninsula even during winter^77^). This indicates a strong homogenization of the MHC pool at a relatively large geographical scale and a minor significance of local adaptation processes in shaping MHC polymorphism in coots. This, in turn, may suggest a relatively strong spatial homogeneity in the pressure of extracellular pathogens on this species.

Population-wide genotyping of key PBR exons in the Eurasian coot indicated that an overall level of polymorphism was similar between MHC-I and MHC-II genes. Although the number of alleles and segregation sites, as well as the total number of mutations were slightly greater at MHC-I, we observed slightly higher nucleotide diversity at the MHC-II. Despite this similarity, our analyses revealed a considerably stronger signature of positive selection and recombination at the MHC-II than MHC-I. At the MHC-I we detected only 8 sites under pervasive positive selection, which was half the number of positively selected sites at the MHC-II (*n* = 17). The rate of non-synonymous to synonymous nucleotide substitutions within the putative PBR sites was also greater within MHC-II, indicating stronger pathogen-driven diversifying selection. Finally, we found a greater number of recombination events and a greater percentage of recombinant sequences at the MHC-II and these recombination mechanisms are known to effectively generate MHC variation under strong pathogen pressure^78^. All these results seem to suggest that, in an evolutionary context, extracellular parasites might have exerted stronger selective pressure on the MHC-II genes in coots, when compared with the intracellular pathogens, whose antigens are recognized by the MHC-I. A similar pattern (i.e. stronger diversifying selection at MHC-II than MHC-I) was previously described in other non-passerine lineages, e.g. Procellariformes^52^ and Phoenicopteriformes^72^, as well as in a large-scale analysis of selection at the non-passerine MHC^27^. An opposite pattern was recorded in passerines, where diversifying selection is usually stronger at the MHC-I^27^. In general, evolutionary trajectories of MHC-I and MHC-II genes in passerine and non-passerine birds may differ, suggesting a contrasting pressure by extra- and intracellular pathogens^27^. As the diversity of ecological niches for extracellular parasites should increase along with the structural size of their hosts^79^, these differences could possibly be linked to greater body sizes of non-passerines, which are thus likely to interact with more diverse faunas of extracellular parasites.

Finally, it is important to acknowledge that research on genes responsible for pathogen recognition in wild non-model animal species, including birds, may not only improve our understanding of disease transmission within natural populations, but may also provide insights into zoonotic transmissions to farm animals and humans. Wild birds are a major reservoir of many zoonotic pathogens (e.g. West Nile virus, influenza A virus, *Campylobacter* and *Salmonella* bacteria)^80^ and can transmit them over long distances and diverse habitats (including urban landscape) during migration^81^. Also, distribution and ecology of many pathogens and their animal hosts is altering with human population growth, urbanisation and environmental changes^82^. These changes are often unpredictable, bringing humans into direct contact with pathogens, which were previously of marginal importance for humankind^82^. Considering the fact that the MHC is intimately linked to immune responses through antigen presentation, it is pivotal in the pathogenesis of many infectious agents and MHC diversity within natural populations should primarily reflect selection from local pathogens and parasites^83^. Thus, a long-term monitoring of temporal changes in MHC allelic composition in wild animals could possibly allow tracking changes in the composition of pathogen faunas and detecting emerging zoonotic diseases.

To sum up, a combination of complementary molecular approaches, i.e. targeted exon genotyping via high-throughput sequencing and de novo genome assembly, allowed us to obtain a high-quality resolution of MHC polymorphism in a non-model bird species from rail family. Our study provided novel insights into the evolution of key antigen-presenting genes of the adaptive immunity in a poorly researched lineage of birds and revealed some unique features of the MHC in our study species (extraordinary duplication rate and allelic richness). Finally, our comparisons of selection and recombination processes clearly indicated that the polymorphism of MHC-I and MHC-II genes in birds may be governed by distinct mechanisms, thereby providing evidence for variation in the evolutionary trajectories of the two MHC classes. While our targeted MHC sequencing focused on a single exon per class, we recommend that future research on the MHC polymorphism in non-model organisms should focus on the entire peptide-binding region (two exons per class), which could provide novel insights into the mechanisms governing MHC diversity in natural populations.

## Supplementary Information

Supplementary Information.

## Data Availability

Raw data have been deposited in GenBank (whole genome shotgun sequences: JABXFB010000001-JABXFB010031348; whole genome shotgun scaffolds: MU065399-MU069429; whole genome assembly: GCA_013372525.1; high-throughput MHC-I sequences: TBA; novel high-throughput MHC-II sequences: MT74863–MT748786).
