# Supplementary material for: Using de novo genome assembly and high-throughput sequencing to characterize the MHC region in a non-model bird, the Eurasian coot
Source: Sci Rep. 2022 Apr 29;12:7031. doi: 10.1038/s41598-022-11018-w (PMC9054815; doi:10.1038/s41598-022-11018-w)
Supplement: Supplementary file 1 — Supplementary Information. [file 41598_2022_11018_MOESM1_ESM.pdf]

Figure S1. Alignments of amino acid MHC-I (A), MHC-IIA (B), and MHC-IIB (C) sequences of the Eurasian coot *Fulica atra*, as retrieved from *de novo* genome assembly. Four randomly selected MHC-I haplotypes were shown. Sequences from the mallard *Anas platyrhynchos* (MH218846 for MHC-I, HM070250 for MHC-IIA), chicken *Gallus gallus* (AF459830 for MHC-I), crested ibis *Nipponia nippon* (AB872444 for MHC-IIA and MHC-IIB), and Chinese egret *Egretta eulophotes* (KC282841 for MHC-IIB) were added for reference.

A

EXON 1		
	**Consensus**	**MGLDRALGLLLLLRVLGWAAC**
	Fulica atra hap*01	.WPL........-WL..R...
	Fulica atra hap*02	....
	Fulica atra hap*03	...........M.........
	Fulica atra hap*04	....
	Anas platyrhynchos	..GALG.V.G...G...G..S
	Gallus gallus	..PCGA...G...AAVCG..A

EXON 2		
	**Consensus**	**ELHSLRYFHTAVSEPSPGVPQFVSMGYVDGNLISRYDSEKGRAVPRADWMAANLDQQHWDQQTQIEQTNQEIYTVNLDTLQSRYNQSG**
	Fulica atra hap*01	V....H..DIG.........E........E...V....NTRWM.................W...NG.N..QMNH.T.AK..E.....S
	Fulica atra hap*02	........................V........V..........S...........................S...............
	Fulica atra hap*03	.......L..............................DTR.M.......KD...................T................
	Fulica atra hap*04	........DI..........E.MF.........V...........................E..VG.N....................
	Anas platyrhynchos	.P......E....D.......Y.AV.....EAFTY....T..TE..V..I..HT......MA.E.F.DTEQN.RM.....RE.....R
	Gallus gallus	...T...IR..MTD....L.W..DV.....E.FMH.N.TAR.....TE.I...T......RE...V.GSEQ.NRE...I.RR....T.

EXON 3		
	**Consensus**	**GAHTLQSMVGCDLLEDNSTRGYWQIAYDGRDFIAFDMDTMTFTAADAAAQITKRNWEADGTVAEHMKQYLENTCPEWLRKYVSYGQAVLERK**
	Fulica atra hap*01	R...W...L.....K............N..........................K..M......D.............K.............
	Fulica atra hap*02	.T.....I.S...............................L............I....R.PIK...D....I.....M..M........K.
	Fulica atra hap*03	.......R.S.........S....V................................VN...V....D..GS......M.............
	Fulica atra hap*04	....Q.H.Y............F.....................V.....E..............SW.Y...S..TT...........M....
	Anas platyrhynchos	.SY.V.V.Y.......G.I..FD.HS.N.K....LYK..L.Y.....G......K..E......RR.Y......I..........KD....R
	Gallus gallus	.S..V.W.S...I...GTI...H.A.......V...KG...L...VPE.VP...K..EG.Y-..GL.....E..V....R..E..K.E.G.R

EXON 4		
	**Consensus**	**EPPTVRVSKKETQGILTLHCRAYGFYPRPITISWLKDGEVRDQETQRGSIVPNSDGTYYTWVSIEAPPGEQDKYRCRVEHASLAEPGVFAW**
	Fulica atra hap*01	...M..A.G..................................K.EQ..........F...............................V.
	Fulica atra hap*02	.......L......N...............A...........H...Q..........F.................................
	Fulica atra hap*03	.....Q..G...N..........V.......................V.
	Fulica atra hap*04	.......L............C..T.........................
	Anas platyrhynchos	.R.E.Q..GM.ADK....S...H.......S.......M.QE...K...T........HI.AT.DVL...R...Q........PQ..L.S.
	Gallus gallus	.R.E...WG..AD.....S...H.......VV......A..G.DA.S.GIV..G....H...T.D.Q..DG...Q........PQ..LYS.

EXON 5		
	**Consensus**	**EPESNSLTIVLWVVV-PLAILAVIAITGFIIWKRRS**
	Fulica atra hap*01	......SS..P......................C..
	Fulica atra hap*02	..D..K....P.............P...........
	Fulica atra hap*03	.S.........G........................
	Fulica atra hap*04	.....Q.....G..............I..V......
	Anas platyrhynchos	K.Q..LIP..AG.A.AVV.VI.AL.--.LAV..SKQ
	Gallus gallus	T.QP.LVP..AG.A.AIV..AI.VGV-....YR.HA

B

Exon	Sequence	Alignment
**EXON 1**	**Consensus**	**M A G G R G V P L V L L A V L A L Q G S G A V K**
	Fulica atra	. . . . S . . . . .
	Anas platyrhynchos	. . T . . . .
	Nipponia nippon	. . . . . . I . . A . . . . . T . R . A . . . .
**EXON 2**	**Consensus**	**V G N R H T Q T D M Y Q R D E R - - - Q Q Q E G G Q F M F D F D G D E I F H V D L Q K K E T I W R L P E F G K F T S F E A Q G A L Q N I A V A K Q N M E I M M K K S N R S Q G T I**
	Fulica atra	. . . . H . . . . P Q Q . . . . . . E . . M . . . . V . . . S . . . . G . . . V . T . . H . . . V S . . Q N . . . . . A .
	Anas platyrhynchos	. . H V L M . . E F . . . . . T - - - R N K . . . . E . S . . . . I . T T . . . . D . . . . R . . . .
	Nipponia nippon	. . . A I I . . . L . . . . . . - - - L . . . . Q . . . . A . . . . M . . . L K . . T E N . . H . . A . .
**EXON 3**	**Consensus**	**A P P E V T V F S E D P V E L G D P N V L I C Y V D K F W P S V I S I T W L K N G Q E V T D G V L E T V F Y R W P D N S F R K F S Y L P F I P T R G D Y Y D C R V E H E G L S S T L T K H W**
	Fulica atra	. . . . M A . . P L . T . . . . N . . . . K . . . . S . . . P . . . . T . . . . H . . . . - - . . . F . . . .
	Anas platyrhynchos	. . . . I . . . . T . . . M . . . . E . . S . . . . . . Q A . . G . Y . . . . W . . R E P . M T . .
	Nipponia nippon	. S . . . . T . . . . R . . . . G Q . C T . . . . A . . . . P T A . L . . .
**EXON 4**	**Consensus**	**E P Q V P L P V S E S T E T L V C A L G L A V G I V G I I V G T I L I I K A M K M N S A R N Q R G L L**
	Fulica atra	. . . . . P . . . . . . Q . . . . I . . . . . I . . F . . . K Q . T . I . T . . . V .
	Anas platyrhynchos	. . . . V . . . . N . . . .
	Nipponia nippon	. . . .

C

Exon	Sequence	Alignment
**EXON 1**	**Consensus**	**M E T G R V L G A G A V L V A L V V L G A H P A C G E E T S**
	Fulica atra	. . . . K . . P
	Egretta eulophotes	. . . . Q . K . . . . .
	Nipponia nippon	. . . . D V . R . . . . .
**EXON 2**	**Consensus**	**G V F Q E L S K S E C Q Y L N G T E R V R Y V A R F I Y N R E Q F A H F D S D V G L Y V G D N P L G E P S A K Y W N S Q P D E L E Q R R A E V D R F C R H N Y G V V T P F T V E R R**
	Fulica atra	. F . . F V F . . . . E . I . . . . V . . . . Y . . . . . A W . . R . . . . . . E . . G . . . . A . . . . . . N . . E A . . . .
	Egretta eulophotes	. . . . . F . R F . . . . L . H . Y . . . . . . L T . . . . A . S . . . . . T . . . . L . . G . . . . V . . . . A . . . . . D . .
	Nipponia nippon	. . . . . . A V Y . . . F . . . . F . E . R . H . . . . Y . . . . P . T . . . . . Q . . . . I . . . A . . . . . . Y . . . . S . . . I . . . .
**EXON 3**	**Consensus**	**V Q P K V S V S P M Q S S S L P Q T D R L V C H V T G F Y P A E I E V K W F K N G Q E E T E R V V S T D V I Q N G D W T Y Q V L V M L E T T P Q R G D T Y M C Q V E H A S L Q H P V T R L W**
	Fulica atra	. . . . L . . F . . . . E . . . . S . . . . K . . . . H . . . . E . . . . M . . . . H . . . .
	Egretta eulophotes	. . . . . R . . . . . . G . . . . . N . . . . Y . . . . Q . . . . . R
	Nipponia nippon	. . . . . K . . . . A . . . . T . . . . . V . . . . Q D .
**EXON 4**	**Consensus**	**E L Q S D A A R S K M L T G V G G F V L G L I F L A L G L F L Y V R K K**
	Fulica atra	. . . . V . . V F . . . . .
	Egretta eulophotes	. V . . . .
	Nipponia nippon	. . . P . . . .
